# Presence of symptoms predicts poor survival and correlates with COX-2 expression in resected intrahepatic cholangiocarcinoma patients

**DOI:** 10.1097/MD.0000000000046715

**Published:** 2025-12-19

**Authors:** Lu Yang, Yingying Wang, Haijing Zheng, Zhaolong Pan, Zezheng Xu, Yubo Wang, Dongyang Li, Yu Wang, Bo Yang, Fenglin Zang, Qin Zhang, Guangtai Cao, Huikai Li, Yunlong Cui, Qiang Wu, Tianqiang Song, Qiang Li, Wei Zhang

**Affiliations:** a Department of Hepatobiliary Surgery, Liver Cancer Prevention and Treatment Research Center, Tianjin Medical University Cancer Institute and Hospital, National Clinical Research Center for Cancer, Tianjin’s Clinical Research Center for Cancer, Tianjin Key Laboratory of Digestive Cancer, Key Laboratory of Cancer Prevention and Therapy, Tianjin, China; b Department of Radiation Oncology, Peking University International Hospital, Beijing, China; c Department of Pathology, Tianjin Medical University Cancer Institute and Hospital; Liver Cancer Center, Tianjin Medical University Cancer Institute and Hospital, National Clinical Research Center for Cancer, Key Laboratory of Cancer Prevention and Therapy, Tianjin’s Clinical Research Center for Cancer, Tianjin, China.

**Keywords:** COX-2, intrahepatic cholangiocarcinoma, prognosis, symptoms

## Abstract

A substantial proportion of patients with intrahepatic cholangiocarcinoma (ICC) present with symptoms such as fever or abdominal pain, which are frequently linked to unfavorable prognostic outcomes. A retrospective analysis of the clinical data from 125 patients diagnosed with ICC was conducted. Univariate and multivariate analyses were carried out to assess overall survival (OS) and disease-free survival (DFS). Subsequently, the patients were stratified into 2 groups based on the presence or absence of symptoms. The presence of symptoms is recognized as a risk factor associated with an unfavorable prognosis (OS: 2.026 [1.133–3.624], *P* = .017); DFS: 1.590 [1.004–2.517], *P* = .048). Patients presenting symptoms exhibit a higher prevalence of lymph node metastasis (*P* = .032), microvascular invasion (*P* = .041), and higher CA19-9 levels (*P* = .020). Survival curves revealed that OS (*P* = .010) and DFS (*P* = .017) were significantly poorer among patients with ICC exhibiting COX-2 (+). Moreover, an analysis of the immune microenvironment in patients with COX-2 (+) indicated a higher presence of CD163+ cells (*P* = .018) compared to that of patients with COX-2 (−). ICC patients with symptoms are associated with a dismal prognosis and a pronounced expression of COX-2 within the tumor tissues. Our study suggests that preoperative symptoms may serve as a clinical indicator of aggressive tumor biology and immune microenvironment alterations, and that COX-2 expression may represent a potential therapeutic target.

## 1. Introduction

Intrahepatic cholangiocarcinoma (ICC) is the second most common primary liver malignancy following hepatocellular carcinoma.^[1]^ Studies have shown that ICC accounts for 10% to 15% of primary liver cancers.^[2]^ ICC has attracted increasing attention over the past 10 to 20 years, primarily due to its morbidity and mortality worldwide.^[3]^ Surgical resection is the only radical treatment. However, radical resection is available in only 20% to 30% of patients due to its asymptomaticity in early stages and patients are often diagnosed in the middle and late stages.^[4]^ Besides, because of its highly aggressive and metastatic nature, ICC has a very high recurrence rate after surgery, ranging from 40% to 80%,^[5]^ and a low 5-year overall survival rate after resection, ranging from 25% to 40%.^[6]^ In clinical practice, a number of patients with ICC have symptoms such as fever and abdominal pain. Although preoperative symptoms such as fever have been reported to be associated with reduced survival in patients with ICC,^[7]^ the generalizability of these findings is limited by variations in population, geography, etiology, and patient characteristics.

The occurrence of symptoms is closely related to prostaglandin E_2_ (PGE_2_), PGE_2_ is an inflammatory mediator that induces fever and abdominal pain.^[8]^ COX-2 is a key enzyme in prostaglandin synthesis. Furthermore, the inflammatory microenvironment may also contribute to the systemic inflammation.^[9]^ According to studies, cancer-associated fibroblasts, type 2 macrophages (M2), and cancer cells also release COX-2 into the tumor microenvironment, causing symptoms such as pain and fever.^[10]^ Previous reports have indicated that COX-2 is an independent risk factor for a poor prognosis in ICC.^[11]^

Although preoperative symptoms and COX-2 expression have been individually associated with poor outcomes in ICC, their interrelationship and combined impact on the tumor immune microenvironment remain poorly characterized. The purpose of this study is to investigate the clinicopathological features and prognosis of symptomatic ICC patients, to analyze the association between symptoms and COX-2 expression, and to explore the relationship between COX-2 and the tumor immune microenvironment.

## 2. Methods

### 2.1. Patients and clinicopathological factors

This retrospective study analyzed 139 consecutive patients with ICC who underwent hepatectomy at Tianjin Medical University Cancer Institute and Hospital between January 2011 and December 2017. After excluding 14 patients (including 11 cases lost to follow-up and 3 cases received non-R0 resection), 56 patients with ICC presenting preoperative symptoms and 69 patients with ICC without symptoms were used to compare the clinicopathological characteristics and survival analysis. All patients underwent curative resection for ICC, defined as complete removal of the tumor and dissection of the hilar and hepatoduodenal ligament lymph nodes, without macroscopic tumor thrombus.^[12]^ In general, resectable intrahepatic tumors were treated with hepatectomy with 1 to 2 cm of surrounding liver tissue. The enrollment criteria for all patients in this study were as follows: over 18 years old; diagnosis of ICC by 2 pathologists; no distant metastasis before radical surgery; no cancer-related treatments before surgery; availability of complete clinicopathological data; and Child-Pugh A or B.

All tumors of ICC were staged according to the tumor, node, metastasis classification system of the International Union Against Cancer (8th edition) and the AJCC (8th edition).^[13]^ Liver function was assessed using the Child-Pugh classification. The Medical Ethics Committee of Tianjin Medical University Cancer Institute and Hospital approved this study, and informed consent was obtained from all patients.

### 2.2. Definition of symptoms

The presence of preoperative symptoms (e.g., fever, abdominal pain) was determined through retrospective review of electronic medical records. Fever was defined as a body temperature ≥ 37.5°C; abdominal pain was defined as persistent or episodic pain requiring medical attention or analgesic use. The duration and severity of symptoms were not systematically recorded due to the retrospective nature of the study.

### 2.3. Immunohistochemistry

Tissue microarrays consisting of 2 mm cores of FFPE tumor tissue were constructed for various staining purposes by selecting representative tumor regions and typical peritumor regions from each case. After dewaxing and rehydrating tissue microarray sections in xylene and gradient ethanol, respectively. After antigen recovery and blocking of endogenous peroxidase activity, sections were sequentially incubated with primary antibody (14 hours at 4°C and 1 hour at 37°C) and HRP-conjugated secondary antibody (1 hour at 37°C). The sections were then color developed with 3, 3′-diaminobenzidine (ZLI-9017; Zhongshan Jinqiao, Beijing, China) for 5 min and counterstained with hematoxylin. Appropriate internal or external positive and negative controls were designed and used in each round. Primary antibodies used in this study were as follows: CD4 (ab133616; dilution 1:500; Abcam, Cambridge, United Kingdom), CD8 (ab17147; dilution 1:50; Abcam), CD163 (ab74604; dilution 1:25; Abcam), and CD66b (ab197678; dilution 1:1000; Abcam), CD68 (T55756F; dilution 1:250; Abmart, Shanghai, China), FOXP3 (ab215206; Abcam), PD-L1 (ab205921; dilution 1:1000; Abcam), and COX-2 (ab283574; dilution 1:250; Abcam). Negative and less than 5% COX-2 positivity were defined as low expression, and greater than or equal to 5% COX-2 positivity was defined as high expression. The threshold for PD-L1 was 1%, with less than or equal to 1% being negative and greater than 1% being positive. CD4+, CD8+ T lymphocytes, CD163+ macrophages, and CD66b+ neutrophils were counted in four 20× fields of view, and the average number of positive cells was taken. The optimal threshold for dichotomizing immune cell infiltration (CD4+, CD8+, CD163+, CD66b+) into “high” and “low” groups was determined using receiver operating characteristic curve analysis, with patient overall survival as the classification variable. The cutoff value corresponding to the maximum Youden’s Index (Sensitivity + Specificity − 1) was selected. The intensity of FOXP3 nuclear staining was multiplied by the percentage of positive cells. A result of 1% or below was classified as low expression, whereas a result exceeding 1% was categorized as high expression. The positivity rate for CD68+ macrophage staining that is less than or equal to 30% is classified as low expression, whereas a rate exceeding 30% is characterized as high expression.

### 2.4. Follow-up and treatment of tumor recurrence

Survival data, including overall survival (OS) and disease-free survival (DFS), were collected until December 30, 2019. Data were censored at the last follow-up for surviving patients. Overall survival was defined as the time from surgery to death from any cause. DFS was defined as the time interval from surgery to first recurrence. Patients were followed up every 3 months for the first 2 years and every 4 to 6 months thereafter, and tests included serum carcinoembryonic antigen, carbohydrate antigen19-9 (CA19-9), liver ultrasonography, computed tomography, and magnetic resonance imaging. If recurrence was confirmed, a second hepatectomy, radiofrequency ablation, percutaneous ethanol injection, transcatheter arterial chemoembolization, external radiotherapy, or symptomatic treatment was performed depending on the number, size, and location of the recurrent tumors.

### 2.5. Statistical analysis

SPSS 27.0 was used for statistical analysis. Student *t* test was used for continuous variables with normal distribution, Mann–Whitney *U* test was used for continuous variables with non-normal distribution, and basic clinical indicators were analyzed by Chi-square test or Fisher’s exact test for categorical variables. Kaplan–Meier survival curve and log-rank test were used to analyze OS and DFS, and COX regression model was used for multivariate analysis. The significant variables with *P* < .05 in univariate analyses were used in the multivariate analyses. A *P* value < .05 was considered statistically significant; NS indicates no significant difference. * indicates that *P* < .05, ** indicates that *P* < .01, and *** indicates that *P* < .001.

## 3. Results

### 3.1. Symptoms was an independent risk factor for DFS and OS

**Figure 1.**
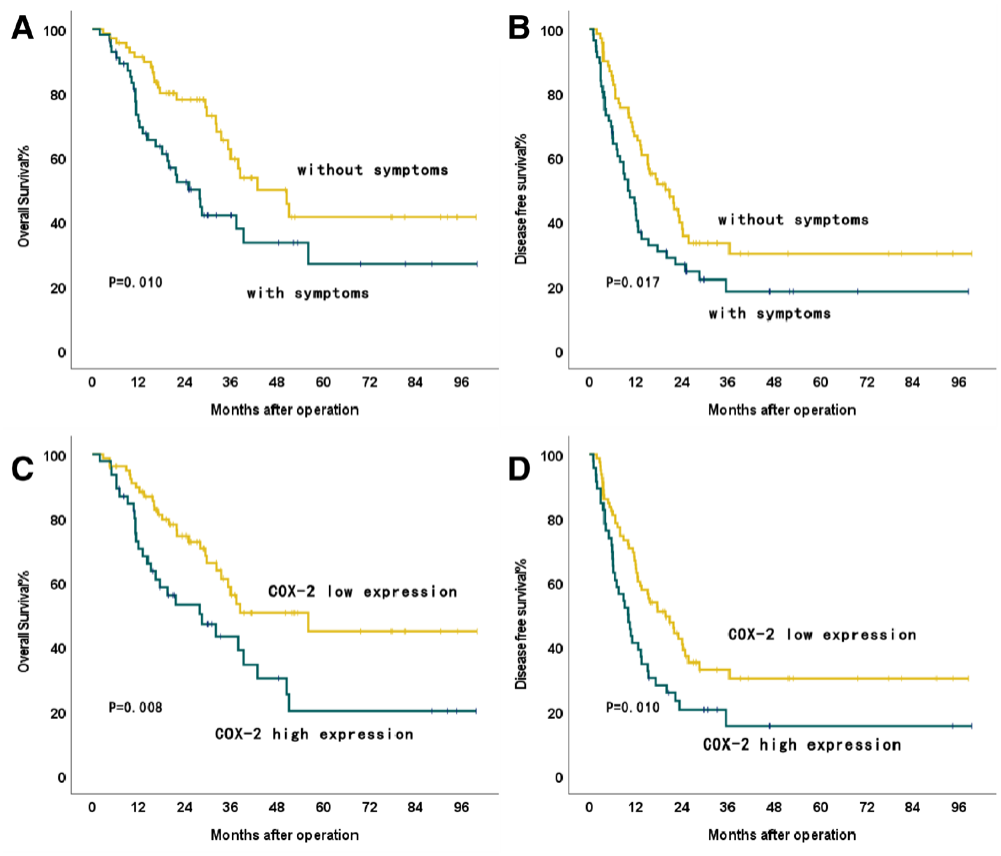
OS and DFS for ICC patients after curative resection. (A) Comparison of OS between patients with and without symptoms. (B) Comparison of DFS between patients with and without symptoms. (C) Comparison of OS between COX-2 negative and positive patients. (D) Comparison of DFS between COX-2 negative and positive patients. DFS = disease-free survival, ICC = intrahepatic cholangiocarcinoma, OS = overall survival.

In the study, the median follow-up duration was 34 months. At the last follow-up, it was observed that 76.8% (43/56) of patients with symptoms experienced recurrence, while 55.4% (31/56) had deceased. The 1- and 3-year DFS rates of symptomatic patients were 44.0% and 18.4%, respectively, in contrast to 66.7% and 33.5% of asymptomatic patients (*P* = .018). Similarly, the 1-, 3-, and 5-year OS rates for symptomatic patients were 71.4%, 42.0%, and 26.9%, respectively, compared to 91.1%, 59.6%, and 41.5% for asymptomatic patients (*P* = .010). Kaplan–Meier analysis demonstrated significantly worse DFS and OS in symptomatic patients compared to asymptomatic patients (median OS: 25.0 vs 42.9 months; *P* = .010; Fig. 1A. median DFS: 10.0 vs 19.7 months; *P* = .017; Fig. 1B).

In the multivariate Cox regression analyses, multiple tumor number, CA19-9 levels ≥ 39 U/mL, alanine aminotransferase levels ≥ 39 U/mL, presence of symptoms, and tumor, node, metastasis stage Ⅲ/Ⅳ were identified as independent risk factors for OS (Table 1). Similarly, the presence of multiple tumors, presence of symptoms, and TNM stage Ⅲ/Ⅳ were established as independent risk factors for DFS (Table 2). The presence of symptoms is recognized as a risk factor associated with an unfavorable prognosis (OS: hazard ratio = 2.026, 95% confidence interval [CI]: 1.133–3.624, *P* = .017; DFS: hazard ratio = 1.590, 95% CI: 1.004–2.517, *P* = .048). Given that the *P* value of DFS multivariate analysis is of borderline significance, the association should be interpreted with caution, and validation in a larger, multicenter cohort is needed to confirm this finding.

**Table 1:** Univariate and multivariate analysis for OS.

Overall survival	Univariate	Multivariate	
Variable	*P* value	*P* value	Hazard ratio (95% CI)
Gender (male vs female)	.226		
Age (>54 vs ≤ 54 yr)	.139		
Number (multiple vs solitary)	<.001	.006	2.759 (1.336–5.695)
Size (>5 vs ≤ 5 cm)	.330		
Microvascular invasion (positive vs negative)	.193		
Child Pugh (B vs A)	.006	.285	0.569 (0.202–1.599)
Cirrhosis (yes vs no)	.302		
Ascites (yes vs no)	.531		
HBeAg (positive vs negative)	.249		
AFP (>20 vs ≤ 20 ng/mL)	.977		
CA19-9 (≥39 vs < 39 U/mL)	<.001	.024	1.978 (1.092–3.582)
ALT (≥39 vs < 39 U/mL)	<.001	.001	2.933 (1.523–5.648)
Symptoms (yes vs no)	.010	.017	2.026 (1.133–3.624)
COX-2 (high vs low)	.008	.513	1.214 (0.678–2.174)
TNM (Ⅲ/Ⅳ vs Ⅰ/Ⅱ)	.013	.013	2.066 (1.169–3.653)

AFP = alpha-fetoprotein, ALT = alanine aminotransferase, OS = overall survival, TNM = tumor, node, metastasis.

**Table 2:** Univariate and multivariate analysis for DFS.

Disease free survival	Univariate	Multivariate	
Variable	*P* value	*P* value	Hazard ratio (95% CI)
Gender (male vs female)	.260		
Age (>54 vs ≤54 years)	.139		
Number (multiple vs solitary)	.012	.003	2.483 (1.364–4.517)
Size (>5 vs ≤5 cm)	.051		
Microvascular invasion (positive vs negative)	.038	.527	1.159 (0.734–1.833)
Child Pugh (B vs A)	.213		
Cirrhosis (yes vs no)	.979		
Ascites (yes vs no)	.124		
HBeAg (positive vs negative)	.589		
AFP (>20 vs ≤ 20 ng/mL)	.269		
CA19-9 (≥39 vs <39 U/mL)	<.001	.051	1.611 (0.997–2.603)
ALT (≥39 vs <39 U/mL)	.233		
Symptoms (yes vs no)	.017	.048	1.590 (1.004–2.517)
COX-2 (high vs low)	.011	.326	1.265 (0.791–2.023)
TNM (Ⅲ/Ⅳ vs Ⅰ/Ⅱ)	.013	<.001	2.196 (1.401–3.442)

AFP = alpha-fetoprotein, ALT = alanine aminotransferase, DFS = disease-free survival, TNM = tumor, node, metastasis.

### 3.2. Comparison of clinicopathological factors and tumor immune microenvironment between ICC patients with and without symptoms

A comparative analysis of clinicopathological data between symptomatic and asymptomatic patients was conducted. Patients with symptoms exhibited higher rates of lymph node metastasis (30.4% vs 14.5%; *P* = .032) and microvascular invasion (MiVI) (58.9% vs 40.6%; *P* = .041). They also demonstrated elevated levels of CA19-9 (68.16 [19.73, 1244] vs 23.95 [11.9, 84.06], *P* = .020), increased gamma-glutamyl transpeptidase (γGT) (73.0 [42.5, 154.25] vs 44.0 [28.0, 107.5], *P* = .009), and alkaline phosphatase (ALP) (112.0 [84.5, 197.75] vs 94.0 [80.0, 125.0], *P* = .014), as well as higher systemic immune-inflammation index (591.7 [362.7, 864.9] vs 430.7 [287.5, 612.7], *P* = .014) and platelet-to-lymphocyte ratio (147.18 [99.5, 201.9] vs 111.7 [82.8, 148.0], *P* = .003) (Table 3).

**Table 3:** Comparison of clinicopathological factors between patients with and without symptoms.

	With symptoms (n = 56)	Without symptoms (n = 69)	*P* value
Gender (male vs female)	28/28 (50.0%)	41/28 (59.4%)	.292
Age (yr) (mean ± SD)	56.6 ± 11.0	58.4 ± 9.0	.310
Tumor size (cm) (median, range)	5 (3.5–8.75)	5 (4–6)	.625
Number (solitary vs multiple)	52/4 (92.9%)	57/12 (82.6%)	.088
TNM (Ⅲ/Ⅳ vs Ⅰ/Ⅱ)	20/36 (35.7%)	20/49 (29.0%)	.423
Lymph node metastasis (positive vs negative)	17/39 (30.4%)	10/59 (14.5%)	.032
Microvascular invasion (positive vs negative)	33/23 (58.9%)	28/41 (40.6%)	.041
CA19-9 (U/mL) (median, range)	68.16 (19.73–1244)	23.95 (11.9–84.06)	.020
HBeAg (positive vs negative)	36/20 (64.3%)	46/23 (66.7%)	.781
Cirrhosis (yes vs no)	15/41 (26.8%)	24/45 (34.8%)	.337
Ascites (yes vs no)	7/49 (12.5%)	2/67 (2.9%)	.086
Child Pugh (B vs A)	5/51 (8.9%)	1/68 (1.4%)	.127
γ-GT (U/L) (median, range)	73 (42.5–154.25)	44 (28–107.5)	.009
ALP (U/L) (median, range)	112 (84.5–197.75)	94 (80–125)	.014
ALT (≥39 vs < 39 U/mL)	23/33 (41.1%)	17/52 (24.6%)	.050
AST (≥40 vs < 40 U/mL)	16/40 (28.6%)	11/58 (15.9%)	.088
PLT (10^9^/L) (mean ± SD)	247.9 ± 78.3	217.6 ± 77.4	.033
Albumin (g/L) (median, range)	43.7 (40.3–46.6)	44.3 (41.0–46.6)	.548
TB (μmol/L) (median, range)	15.3 (11.0–20.7)	13.9 (11.7–17.1)	.377
SII (median, range)	591.7 (362.7–864.9)	430.7 (287.5–612.7)	.014
GPR (median, range)	0.31 (0.18–0.83)	0.28 (0.13–0.53)	.113
APRI (median, range)	0.31 (0.18–0.6)	0.27 (0.2–0.51)	.688
NLR (median, range)	2.29 (1.86–3.13)	2.0 (1.68–2.75)	.177
PLR (median, range)	147.18 (99.5–201.9)	111.7 (82.8–148.0)	.003

ALP = alkaline phosphatase, ALT = alanine aminotransferase, APRI = aspartate aminotransferase to platelet ratio index, CA19-9 = carbohydrate antigen19-9, GPR = gamma-glutamyl transpeptidase [γ-GT]-to-platelet ratio, NLR = neutrophil-to-lymphocyte ratio, PLR = platelet-to-lymphocyte ratio, SII = systemic immune-inflammation index, defined as platelet times neutrophil/lymphocyte, TB = total bilirubin, TNM = tumor, node, metastasis.

**Figure 2.**
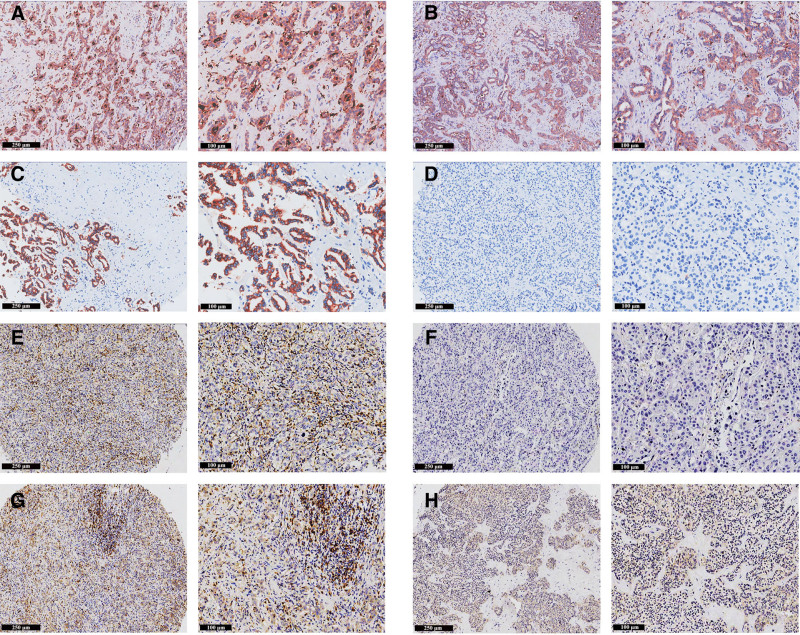
Immune microenvironment in ICC patients with and without symptoms. (A) Negative staining for CD68. (B) Positive staining for CD68. (C) Negative staining for COX-2. (D) Positive staining for COX-2. (E) Negative staining for CD4. (F) Positive staining for CD4. (G) Negative staining for CD8. (H) Positive staining for CD8. Scale bars: 250 μm (100×); 100 μm (200×). ICC = intrahepatic cholangiocarcinoma.

**Figure 3.**
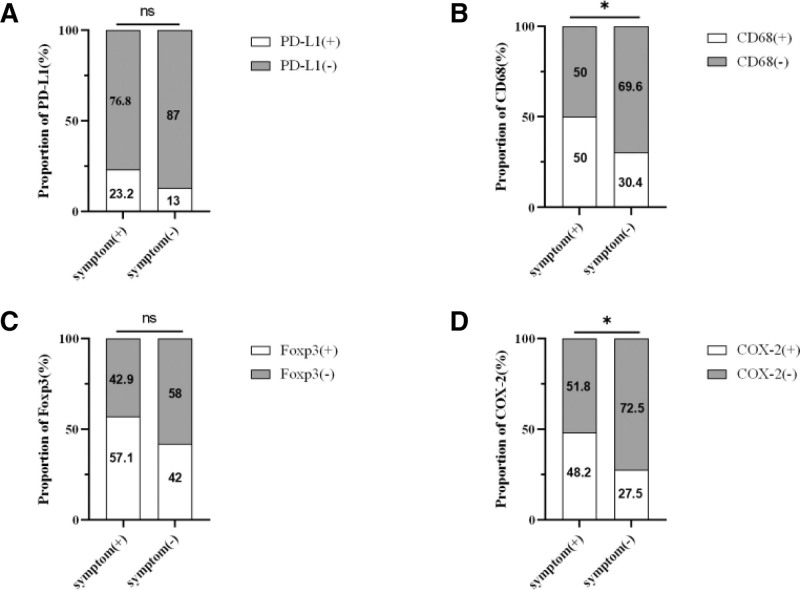
The results of immunohistochemistry between the 2 groups of patients with and without symptoms. (A): PD-L1; (B): CD68; (C): Foxp3; (D): COX-2.

**Figure 4.**
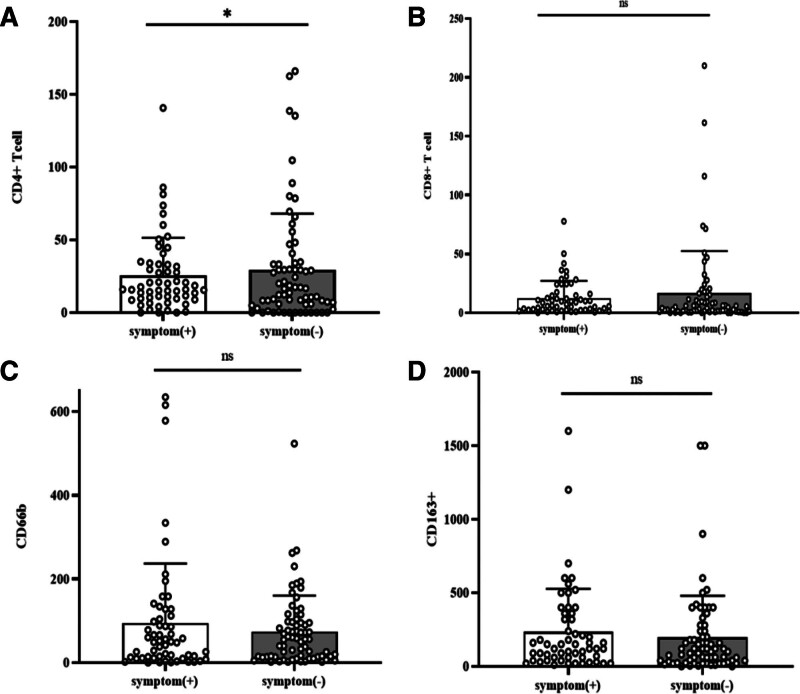
The results of immunohistochemistry between the 2 groups of patients with and without symptoms. (A): CD4+ T cell; (B): CD8+ T cell; (C): CD66b; (D): CD163. The height of the column represents the average value. The error bars represent the standard deviation (SD). Each data point represents one patient. With symptoms (n = 56); Without symptoms (n = 69).

Furthermore, an analysis of the immune microenvironment in patients with and without symptoms was conducted (Figs. 2–4), revealing that patients with symptoms had elevated levels of CD4+ T cells (78.6% vs 59.4%; *P* = .022; Table 4) and CD68+ cells (50.0% vs 30.4%; *P* = .026; Table 4).

**Table 4:** Comparison of immune microenvironment between patients with and without symptoms.

	With symptoms (n = 56)	Without symptoms (n = 69)	*P* value
PD-L1 (positive vs negative)	13/43 (23.2%)	9/60 (13.0%)	.138
CD66b+ (high vs low)	9/47 (16.1%)	9/60 (13.0%)	.632
CD4+ (high vs low)	44/12 (78.6%)	41/28 (59.4%)	.022
CD8+ (high vs low)	12/44 (21.4%)	15/54 (21.7%)	.967
CD163+ (high vs low)	46/10 (82.1%)	51/18 (73.9%)	.272
CD68+ (high vs low)	28/28 (50.0%)	21/48 (30.4%)	.026
FOXP3 (high vs low)	32/24 (57.1%)	29/40 (42.0%)	.093
COX-2 (high vs low)	27/29 (48.2%)	19/50 (27.5%)	.017

### 3.3. Clinical outcomes of patients with ICC with COX-2 (+) and COX-2 (−)

Patients with symptoms exhibited a higher rate of COX-2 (+) in the tumor tissue compared to asymptomatic patients (48.2% vs 27.5%; *P* = .017; Table 4; Fig. 3D). Our investigation also revealed a significant association between COX-2 (+) and DFS and OS. Kaplan–Meier analysis was conducted to compare the clinical outcomes of patients in the two groups. Patients with COX-2 (+) experienced significantly poorer OS and DFS compared to those with COX-2 (−) (median OS, 27.8 vs 38.3 months; *P* = .008; median DFS, 10.0 vs 17.6 months; *P* = .011; (Fig. 1C and D).

### 3.4. Comparison of clinicopathological factors and tumor immune microenvironment between ICC patients with COX-2 (+) and COX-2 (−)

**Figure 5.**
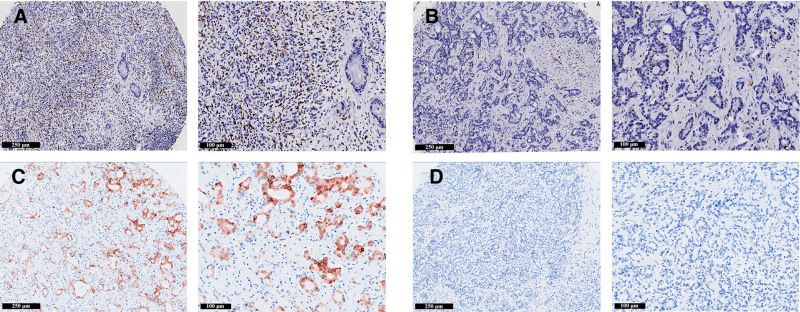
Immune microenvironment staining in ICC patients with and without COX-2. (A) Negative staining for CD163. (B) Positive staining for CD163. (C) Negative staining for COX-2. (D) Positive staining for COX-2. Scale bars: 250 μm (100×); 100 μm (200×). ICC = intrahepatic cholangiocarcinoma.

**Figure 6.**
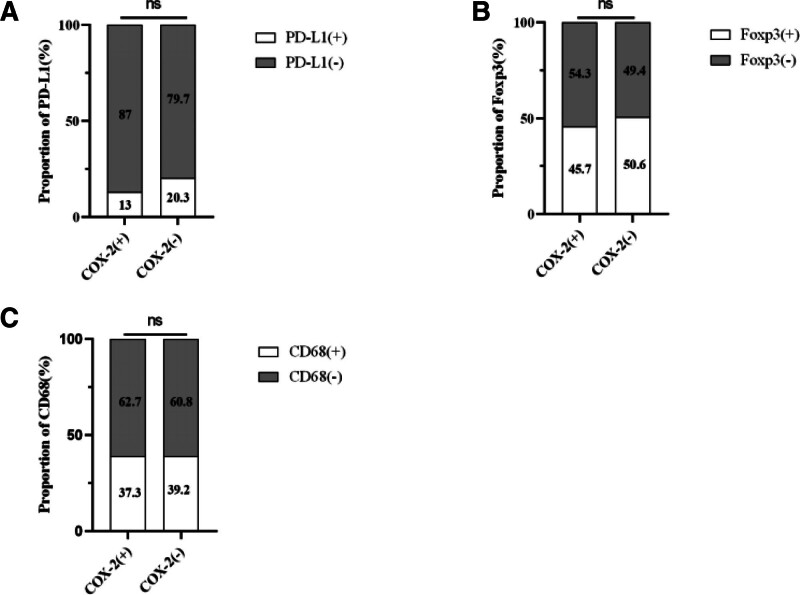
The results of immunohistochemistry between the 2 groups of patients with and without COX-2. (A): PD-L1; (B): Foxp3; (C): CD68.

**Figure 7.**
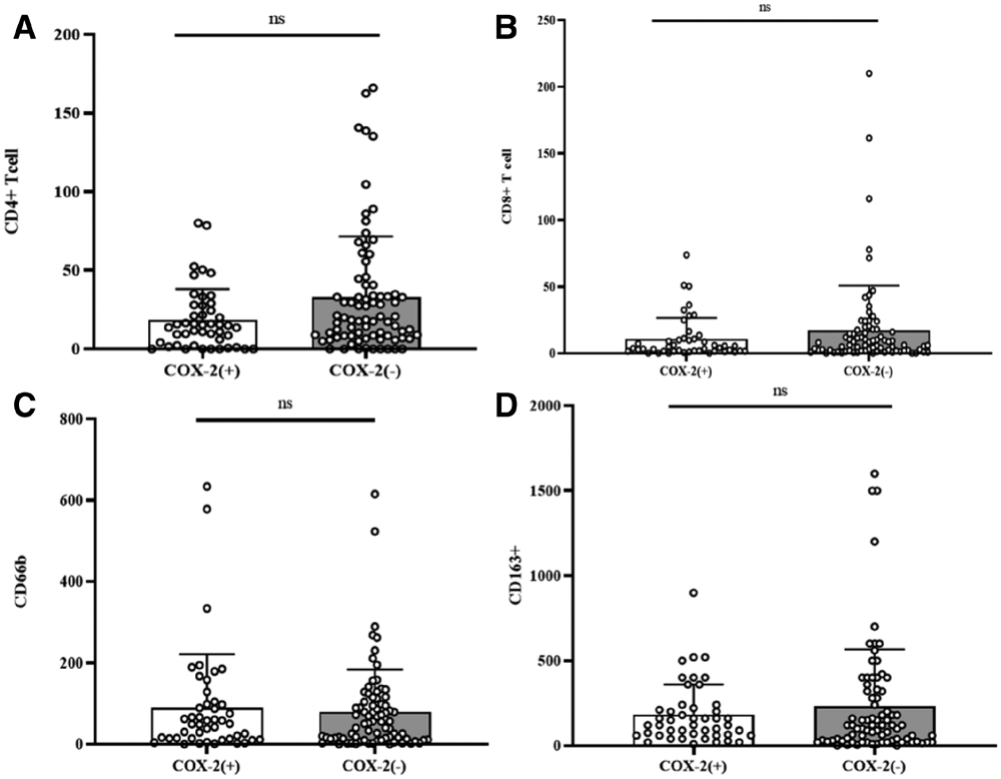
The results of immunohistochemistry between the 2 groups of patients with and without COX-2. (A): CD4+ T cell; (B): CD8+ T cell; (C): CD66b; (D): CD163. The height of the column represents the average value. The error bars represent the standard deviation (SD). Each data point represents one patient. COX-2(+) (n = 46); COX-2(−) (n = 79).

A comparison of clinicopathological data between patients with COX-2 (+) and COX-2 (−) was conducted. Patients with COX-2 (+) demonstrated a higher incidence of lymph node metastasis (34.8% vs 13.9%; *P* = .006), elevated MiVI rates (60.9% vs 41.8%; *P* = .039), and increased serum CA19-9 levels (147.0 [18.8, 1643.3] vs 26.1 [10.3, 68.7], *P* < .001) (Table 5). Additionally, patients with COX-2 (+) exhibited a higher prevalence of ascites (15.2% vs 2.5%; *P* = .022), worse Child-Pugh scores (10.9% vs 1.3%; *P* = .047), elevated γGT levels (88.0 [45.5, 280.5] vs 45.0 [30.0, 93.0], *P* = .002), increased ALP levels (124.5 [85.5, 243.3] vs 94.0 [80.0, 123.0], *P* = .002), higher alanine aminotransferase levels (50% vs 21.5%; *P* < .001), elevated aspartate aminotransferase levels (37.0% vs 12.7%; *P* = .001), and increased total bilirubin levels (16.2 [12.8, 21.9] vs 13.8 [10.8, 17.2], *P* = .011) (Table 5). Patients diagnosed with ICC and exhibiting COX-2 (+) demonstrated elevated gamma-glutamyl transpeptidase-to-platelet ratio (0.43 [0.23, 1.08] vs 0.22 [0.13, 0.47], *P* = .002), increased aspartate aminotransferase-to-platelet ratio index (0.38 [0.19, 0.75] vs 0.26 [0.20, 0.39], *P* = .033), and higher platelet-to-lymphocyte ratio (139.2 [106.8, 174.5] vs 124.2 [80.6, 164.8], *P* = .033) (Table 5). Additionally, an analysis of the immune microenvironment in patients with COX-2 (+) and COX-2 (−) was conducted (Figs. 5–7), revealing that those with COX-2 (+) exhibited elevated CD163+ cells (89.1% vs 70.9%; *P* = .018; Table 6).

**Table 5:** Comparison of clinicopathological factors between patients with and without COX-2.

	COX-2 (+) (n = 46)	COX-2 (−) (n = 79)	*P* value
Gender (male vs female)	24/22 (52.2%)	45/34 (57.0%)	.604
Age (yr) (mean ± SD)	58.3 ± 10.6	57.2 ± 9.7	.571
Tumor size (cm) (median, range)	4.5(4–7)	5 (4–6.5)	.688
Number (solitary vs multiple)	40/6 (87.0%)	69/10 (87.3%)	.951
TNM (Ⅲ/Ⅳ vs Ⅰ/Ⅱ)	18/28 (39.1%)	22/57 (27.8%)	.192
Lymph node metastasis (positive vs negative)	30/16 (34.8%)	68/11 (13.9%)	.006
Microvascular invasion (positive vs negative)	28/18 (60.9%)	33/46 (41.8%)	.039
CA19-9 (U/mL) (median, range)	147.0 (18.8–1643.3)	26.1 (10.3–68.7)	<.001
HBeAg (positive vs negative)	27/19 (58.7%)	55/24 (69.6%)	.215
Cirrhosis (yes vs no)	12/34 (26.1%)	27/52 (34.2%)	.346
Ascites (yes vs no)	7/39 (15.2%)	2/77 (2.5%)	.022
Child Pugh (B vs A)	5/41 (10.9%)	1/78 (1.3%)	.047
γ-GT (U/L) (median, range)	88.0 (45.5–280.5)	45 (30–93)	.002
ALP (U/L) (median, range)	124.5 (85.5–243.3)	94 (80–123)	.002
ALT (≥39 vs < 39 U/mL)	23/23 (50.0%)	17/62 (21.5%)	<.001
AST (≥40 vs < 40 U/mL)	17/29 (37.0%)	10/69 (12.7%)	.001
PLT (10^9^/L) (mean ± SD)	232.7 ± 72.7	230.3 ± 82.8	.871
Albumin (g/L) (median, range)	43.5 (39.3–46.5)	44.2 (41.4–46.6)	.338
TB (μmol/L) (median, range)	16.2 (12.8–21.9)	13.8 (10.8–17.2)	.011
SII (median, range)	489.5 (373.6–708.5)	535.4 (290.9–765.3)	.914
GPR (median, range)	0.43 (0.23–1.08)	0.22 (0.13–0.47)	.002
APRI (median, range)	0.38 (0.19–0.75)	0.26 (0.2–0.39)	.033
NLR (median, range)	2.17 (1.81–2.87)	2.3 (1.7–2.9)	.826
PLR (median, range)	139.2 (106.8–174.5)	124.2 (80.6–164.8)	.033

ALP = alkaline phosphatase, ALT = alanine aminotransferase, APRI = aspartate aminotransferase to platelet ratio index, CA19-9 = carbohydrate antigen19-9, GPR = gamma-glutamyl transpeptidase [γ-GT]-to-platelet ratio, NLR = neutrophil-to-lymphocyte ratio, PLR = platelet-to-lymphocyte ratio, SII = systemic immune-inflammation index, defined as platelet times neutrophil/lymphocyte, TB = total bilirubin, TNM = tumor, node, metastasis.

**Table 6:** Comparison of immune microenvironment between patients with and without COX-2.

	COX-2 (+) (n = 46)	COX-2 (−) (n = 79)	*P* value
PD-L1 (positive vs negative)	6/40 (13.0%)	16/63 (2.3%)	.307
CD66b+ (high vs low)	9/37 (19.6%)	9/70 (11.4%)	.209
CD4+ (high vs low)	31/15 (67.4%)	54/25 (68.4%)	.911
CD8+ (high vs low)	8/38 (17.4%)	19/60 (24.1%)	.383
CD163+ (high vs low)	41/5 (89.1%)	56/23 (7.9%)	.018
CD68+ (high vs low)	18/28 (39.1%)	31/48 (39.2%)	.990
FOXP3 (high vs low)	21/25 (45.7%)	40/39 (5.6%)	.591

## 4. Discussion

Due to the increasing global prevalence of ICC,^[3]^ this disease has gained considerable attention. Various prognostic indicators have been identified, including elevated preoperative CA19-9 levels, multiple tumors, vascular invasion, and lymph node metastasis. Elevated CA19-9 levels often signify liver inflammation, cirrhosis, and a poor prognosis in patients with hepatocellular carcinoma.^[14]^ In our analysis of ICC patients, we found that patients with symptoms and high COX-2 expression also have a worse prognosis.

Several factors may contribute for poor prognosis in ICC patients with symptoms. First, symptomatic patients have a higher rate of lymph node metastasis, MiVI, and higher levels of CA19-9, which indicated aggressive tumor biology. Second, elevated γGT, ALP indicating more severe liver inflammation, it has been reported that liver inflammation contribute to tumor metastasis.^[15]^ The abnormal liver function indicators observed in ICC patients may be attributed to tumor invasion of hepatocytes, leading to the release of liver enzymes into the cytoplasm. This phenomenon can explain the development of ascites and poor liver function. Third, pain may be associated with tumor invasion of the liver envelope or nerves. Fourth, we also find that patients with ICC with fever have higher levels of systemic immune-inflammation index and PLR than those without fever. Tumors release inflammatory cytokines, especially endogenous pyrogen-related cytokines such as interleukin-1, interleukin-6, interleukin-8, and tumor necrosis factor, which cause fever. Fever is associated with inflammatory and immunosuppressive states that impede the adaptive anti-tumor response and lead to tumor progression.^[16]^ Systemic inflammation may indicate immunosuppressive tumor microenvironment. We also found that higher CD68+ cell levels and higher COX-2 levels in the tumor tissue. Hashemi et al^[10]^ found that M2 cells and cancer cells can release COX-2 into the tumor microenvironment, causing symptoms and poor prognosis.

Previous reports have indicated that COX-2 is an independent risk factor for a poor prognosis in ICC.^[11]^ However, in our study, COX-2 is only significant in the univariate analysis of OS and DFS in ICC patients and does not reach significance in the multivariate analysis. This may be due to the stronger influence of symptoms. Numerous studies have demonstrated the involvement of COX-2 in various cancer processes, such as lung, colon, breast, prostate, leukemia, bladder, cervix, neuroblastoma, and head and neck cancers.^[17–21]^ In our study, we find that high expression of COX-2 and symptomatic ICC patients exhibit more aggressive tumor characteristics, including elevated serum CA19-9 levels, higher incidence of lymph node metastasis and microvascular invasion, all of which are linked to aggressive tumor biology. COX-2 functions as an inhibitor of apoptosis and mediates cancer cell growth, tumor migration, tumor invasiveness and metastasis. For example, it promotes blood vessel formation through the vascular endothelial growth factor pathway.^[10,22]^ Increased levels of IL-6, often accompanying COX-2, can promote tumor progression and maintain tumor stemness.^[23]^ In conclusion, COX-2 plays a crucial role in tumorigenesis and development.

COX-2 is closely associated with systematic inflammation, which explains the elevated inflammatory markers such as gamma-glutamyl transpeptidase-to-platelet ratio, aspartate aminotransferase-to-platelet ratio index, and PLR in patients with high COX-2 expression. Tumor cells are known to secrete inflammatory mediators, including chemokines, cytokines, and COX-2, which in turn lead to increased levels of inflammatory markers.^[24]^

The tumor microenvironment of patients with elevated COX-2 expression is examined, revealing a significant increase in CD163+ cells. Tumor-associated macrophages (TAMs) are important inflammatory cells in the tumor microenvironment.^[25]^ TAMs consist of 2 subtypes: M1 and M2.^[26]^ M1-subtype TAMs, known as classically activated macrophages, demonstrate pro-inflammatory and anti-tumor properties, while M2-subtype TAMs, known as alternatively activated macrophages, facilitate tumor growth, angiogenesis, and suppress adaptive immunity.^[27]^ CD68+ and CD163+ are widely used biomarkers for detecting TAMs in tumor specimens. CD68+ identifies the entire TAM population, while CD163+ specifically marks M2 TAMs.^[28]^ Previous studies have shown an association between the infiltration level of M2 TAMs and poor prognosis in patients with various cancers, including pancreatic ductal adenocarcinoma,^[29]^ hepatocellular carcinoma,^[30]^ breast cancer, osteosarcoma,^[31]^ gastric carcinoma,^[32]^ and intrahepatic cholangiocarcinoma.^[33]^ TAM infiltrates into the tumor environment, promotes tumor growth and reduces patient survival.^[34]^ Besides, M2-TAM release COX‐2 which also contribute to tumor invasion, angiogenesis, and metastasis.^[9]^ Consequently, it is hypothesized that the worse prognosis observed in patients with elevated COX-2 expression might be linked to immune suppression within the body, although the exact underlying mechanism necessitates further investigation.

**Figure 8.**
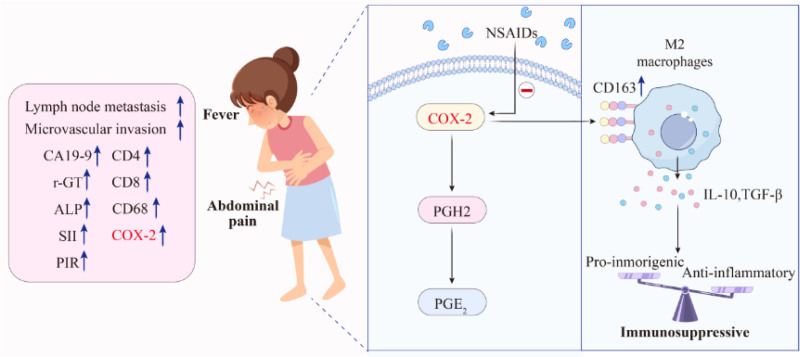
The relationship between COX-2 and symptoms and their respective clinicopathological features and microenvironment, as well as mechanisms of COX-2 inhibitors. AA = arachidonic acid, PGH_2_ = prostaglandin H_2_, PGES = prostaglandin E synthase, PGE_2_ = prostaglandin E_2._

This study identifies a correlation between symptoms and COX-2 expression (Fig. 8). The occurrence of symptoms is closely related to PGE_2_. The phospholipids in the cell membrane are converted to arachidonic acid and released from the cell membrane. After the free arachidonic acid is combined with COX, prostaglandin H_2_ is generated under the catalysis of cyclooxygenase. The unstable prostaglandin H_2_ is eventually transformed into PGE_2_ by the action of specific E type prostaglandin E synthase (PGES). In this process, COX-2 plays a critical role.

Therefore, in cases where patients exhibit symptoms such as fever and abdominal pain prior to surgery, prophylactic administration of COX-2 inhibitors may improve outcomes, but this remains speculative due to the lack of clinical trial data. Therefore prospective clinical trials are urgently needed. Nonsteroidal anti-inflammatory drugs are commonly used to inhibit COX-2, including ibuprofen,^[35]^ sulindac,^[36]^ nimesulide,^[37]^ diclofenac, indomethacin, and aspirin.^[38]^ On the other hand, specific COX-2 inhibitors, such as celecoxib, nimesulide, and meloxicam,^[39]^ are associated with a lower incidence of gastrointestinal adverse reactions.

The current study has several limitations. First, this is a retrospective study from a single institution, which may introduce selection bias and limit the generalizability of the findings, and the sample size was limited in this study, so larger scale, multicenter prospective studies are required to validate these findings. Second, further investigation at the molecular level is required to understand the impact of COX-2 on tumor invasion. Third, due to the retrospective nature of the study, we were unable to fully account for potential confounders such as patient comorbidities, performance status, or detailed medication history, which might influence both the presence of symptoms and overall survival.

## 5. Conclusion

The presence of symptoms and high expression of COX-2 are both considered as prognostic risk factors for ICC patients. Patients with symptoms are more likely to have aggressive tumor biology including lymph node metastasis, microvascular invasion, and have higher levels of CA19-9. Furthermore, Patients with symptoms tend to have poorer liver function and higher levels of systemic inflammation and pro-tumoral inflammation in the tumor microenvironment. COX-2 correlate with presence of symptoms and is also associated with aggressive tumor characteristics. The association between symptoms and COX-2 expression suggests that preoperative administration of COX-2 inhibitors could be a potential strategy to improve the prognosis of symptomatic ICC patients, a hypothesis that requires validation in future prospective studies.

## Author contributions

**Data curation:** Haijing Zheng.

**Investigation:** Yingying Wang.

**Methodology:** Zhaolong Pan, Huikai Li, Yunlong Cui.

**Project administration:** Guangtai Cao, Yunlong Cui.

**Resources:** Fenglin Zang.

**Software:** Qin Zhang.

**Supervision:** Bo Yang, Tianqiang Song, Qiang Li.

**Visualization:** Qiang Wu.

**Writing – original draft:** Lu Yang.

**Writing – review & editing:** Zhaolong Pan, Zezheng Xu, Yubo Wang, Dongyang Li, Yu Wang, Wei Zhang.
